# Canagliflozin promotes osteoblastic MC3T3-E1 differentiation *via* AMPK/RUNX2 and improves bone microarchitecture in type 2 diabetic mice

**DOI:** 10.3389/fendo.2022.1081039

**Published:** 2022-12-16

**Authors:** Peiyang Song, Tianyi Chen, Shunli Rui, Xiaodong Duan, Bo Deng, David G. Armstrong, Yu Ma, Wuquan Deng

**Affiliations:** ^1^ Department of Endocrinology, Chongqing Emergency Medical Center, Chongqing University Central Hospital, School of Medicine, Chongqing University, Chongqing, China; ^2^ Department of Rehabilitation, The Affiliated Hospital of Southwest Medical University, Luzhou, Sichuan, China; ^3^ Department of Surgery, Keck School of Medicine of University of Southern California, Los Angeles, CA, United States

**Keywords:** SGLT2 inhibitors, canagliflozin, bone metabolism, AMPK/RUNX2, type 2 diabetes mellitus

## Abstract

Individuals with type 2 diabetes mellitus (T2DM) have an increased risk of bone metabolic disorders and bone fracture due to disease progression and clinical treatment. The effect of sodium-glucose cotransporter 2 (SGLT2) inhibitors, now greatly prescribed for the treatment of T2DM, on bone metabolism is not clear. This study aimed to explore the possible influence of bone metabolic disorder and the underlying mechanism through a comparison of three different SGLT2 inhibitors (canagliflozin, dapagliflozin, and empagliflozin) in the treatment of type 2 diabetic mice. For the *in vivo* experiments, four groups (DM, DM+Cana, DM+Dapa, and DM+Empa) were established using micro-CT to detect the bone microarchitecture and bone-related parameters. The study results indicated that canagliflozin, but not dapagliflozin or empagliflozin, increased bone mineral density (p<0.05) and improved bone microarchitecture in type 2 diabetic mice. Furthermore, canagliflozin promoted osteoblast differentiation at a concentration of 5 μM under high glucose concentration (HG). Phosphorylated adenosine 5’-monophosphate (AMP)-activated protein kinase (AMPK) α (Thr172) has been confirmed to activate run-related transcription factor-2 (RUNX2) to perform this function. This effect can be partially reversed by the AMPK inhibitor dorsomorphin (compound C) and strengthened by the AMPK activator acadesine (AICAR) *in vitro*. The level trend of RUNX2 and p-AMPK *in vivo* were consistent with those *in vitro.* This study suggested that canagliflozin played a beneficial role in bone metabolism in type 2 diabetic mice compared with dapagliflozin and empagliflozin. It provides some theoretical support for the chosen drugs, especially for patients with osteoporosis or a high risk of fracture.

## 1 Introduction

Type 2 diabetes mellitus (T2DM) leads to osteoporosis and predisposes patients to increased fracture risk through the bone metabolic imbalance between osteoblasts and osteoclasts (1, 2). Bone material properties and bone microarchitecture are altered because of diabetic complications progression and medication utilization (3, 4). Notably, the role of antihyperglycemic agents used for T2DM treatment in bone metabolism is gaining increasing interest. Exploring the effect and the mechanism of bone metabolism during the treatment of a new class of antihyperglycemic agents in T2DM is meaningful for choosing drugs in the clinic.

Some of current medications for type 2 diabetes mellitus, such as metformin, glimepiride, glucagon-like peptide-1 (GLP-1), and insulin, possibly played beneficial roles in bone metabolism (5–8). Canagliflozin, dapagliflozin, and empagliflozin, three sodium-glucose cotransporter 2 (SGLT2) inhibitors have been approved for the treatment of T2DM by promoting urinary glucose excretion in an insulin-independent manner (9–12). The side effect of SGLT2 inhibitors on bone metabolism in patients with T2DM remains controversial (13, 14): the CANVAS study reported canagliflozin possible increased risk of fracture compared to placebo (15), but the CREDENCE study showed no evidence that the fracture risk observed was related to the treatment of canagliflozin (16). Similarly, dapagliflozin does not affect bone formation and resorption markers (17). Empagliflozin did not increase the risk of bone fractures compared with placebo or glimepiride in a 4-year head-to-head study (18). Dapagliflozin showed a therapeutic effect on atherosclerosis in our previous study (19). Therefore, exploring the underlying bone metabolic mechanism with SGLT2i treatment in T2DM is distinctly meaningful.

**Figure 1:**
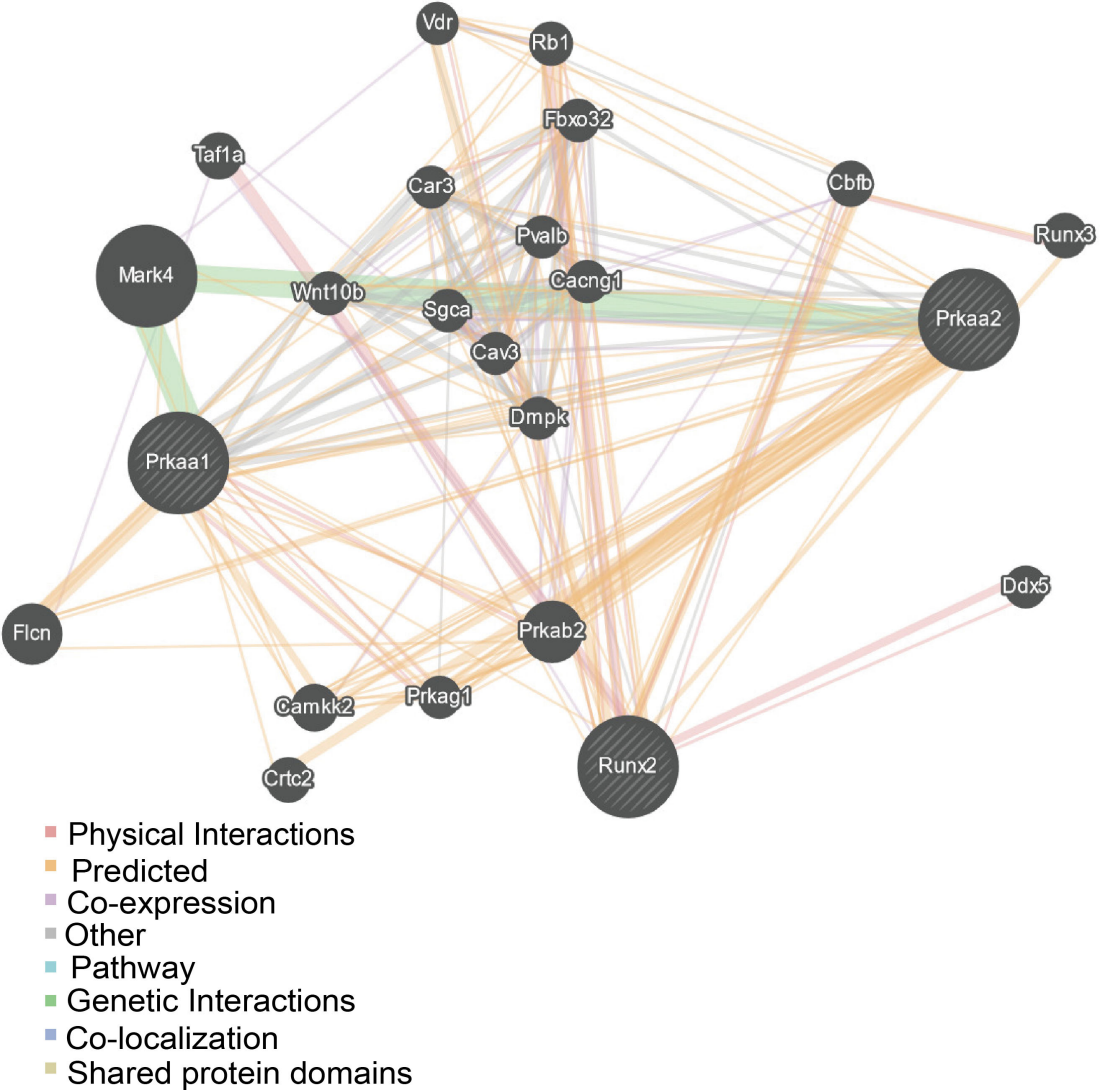
Gene-gene network for the included gene made by the GeneMENIA internet.

DM-related osteoporosis can change gait and balance, making patients prone to falling. Bone quality deteriorates as a result of the accumulated advanced glycation end-products (AGEs) in collagen and dysfunctions of osteoblasts and osteoclasts (20, 21). Activation of the NOTCH, WNT/β-catenin, BMP/Smad, and Hedgehog signaling pathways has been shown to participate in osteogenic differentiation (22). During antidiabetic therapy with metformin, it has been demonstrated that AMP-activated protein kinase (AMPK) plays a vital role in bone metabolism (23–26). Run-related transcription factor-2 (RUNX2), an essential transcription factor in bone formation and a key marker of osteoblast differentiation (27), is a novel substrate of AMP-activated kinase (AMPK), indicating that AMPK signaling may involve in regulating osteogenic differentiation (28–30). Furthermore, we performed a gene interaction network analysis for Prkaa2 (gene ID: 108079), a gene expressing p-AMPK (Thr172), and RUNX2 (gene ID: 12393) through GeneMANIA (http://www.genemania.org/). The prediction results suggested that Prkaa2 had a direct relationship with RUNX2 (**Figure 1**).

Therefore, a comparison of canagliflozin, dapagliflozin, and empagliflozin on bone metabolism in antihyperglycemic therapy is presented here for the first time. It also attempted to elucidate the possible molecular action mechanism of SGLT2 inhibitors *in vivo* and *in vitro* in order to explain the controversial clinical outcomes.

## 2 Materials and methods

### 2.1 Type 2 diabetic mouse model

A total of forty 5-week-old C57/B6 male mice were fed with a 60% high-fat diet for two weeks to induce insulin resistance. The T2DM mouse model was established by intraperitoneal injection of low-dose streptozotocin (STZ) (25 mg/kg) for 5 consecutive days. After 2 weeks, 24 mice with blood glucose levels higher than 16.7 mmol/L were considered to indicate type 2 diabetes mellitus (T2DM). Canagliflozin, dapagliflozin, and empagliflozin (Selleck Chemicals, Houston, TX) were mixed in a 60% high-fat diet separately and fed to T2DM mice. All mice were assigned to as following four groups: 1) T2DM without treatment (DM group); 2) T2DM treated with canagliflozin (100 mg/kg/d) (DM+Cana group); 3) T2DM treated with dapagliflozin (10 mg/kg/d) (DM+Dapa group); and 4) T2DM treated with empagliflozin (10 mg/kg/d) (DM+Empa group). After 10 weeks, femoral tissues and plasma samples were collected. The ethics committee of Chongqing University Central Hospital has approved all animal experiments in accordance with the Declaration of Helsinki.

### 2.2 Micro-CT imaging and analysis

An analysis of bone parameters was performed with a high-resolution micro-CT imaging system (Bruker, SkyScan 1176, USA) and analysis software, including NRecon reconstruction^®^, Bruker CTAn software (RRID: SCR_021338), and CTvol. Specifically, volumetric areas were selected within the endosteal border to analyze the trabecular morphology of the femur (1 mm from the growth plate extending approximately 4 mm).

### 2.3 CCK8 assay

5000 cells per well were seeded into 96-well plates and incubated with CCK8 solution at 37°C for 2 hours. A 450 nm absorbance measurement was performed at the end of the incubation period.

### 2.4 Histological analysis

After being assessed by micro-CT scanning, femurs were fixed with 10% polyoxymethylene, decalcified and embedded in paraffin. Sections were cut into sagittal slides (4 μm thickness). Hematoxylin and eosin (HE) staining and toluidine blue staining were performed according to routine protocols. A panoramic scanner (3D Hitech Ltd.) was used to scan whole slides, and Case Viewer software was used to obtain the image.

### 2.5 Immunofluorescence

The paraffin sections mentioned above were processed for immunofluorescence staining for the detection of RUNX2, osteocalcin (OCN), and p-AMPKα (Thr172), and standard immunofluorescence procedures were followed. The primary antibodies were RUNX2 (Abcam, USA, ab192256), OCN (Santa Cruz Biotechnology Cat# sc-365797, RRID : AB_10859392), and p-AMPKα (Thr172) (Cell Signaling Technology, USA, CST2535). Images were captured by immunofluorescence microscopy.

### 2.6 Cell culture

MC3T3-E1 Subclone 14 cells were purchased from Procell company (Wuhan, China) (RRID : CVCL_5437) and were cultured with α-mem medium containing 10% fetal bovine serum, 1% penicillin-streptomycin and a normal glucose concentration (NG) of 5.5 mM. For high-glucose treatment, the culture medium mentioned contained a 33 mM glucose concentration supplied with D-glucose (Procell company, Wuhan, China), and the cells were treated for 7 days prior to performing the experiments. Cells were seeded on 6-well or 12-well plates in complete medium for 12 h. Canagliflozin (Selleck Chemicals, Houston, TX), a p-AMPK inhibitor dorsomorphin (compound C, Selleck Chemicals, Houston, TX), or an AMPK activator, acadesine (AICAR, Selleck Chemicals, Houston, TX) was added and removed after incubation for 12 h. Then, MC3T3-E1 cells were induced with osteogenic inductive medium (Procell company, Wuhan, China) on the indicated days to perform ALP staining, Alizarin red staining, and protein collection.

### 2.7 ICC

Cells were seeded in 6-well plates plated with glass slides. After a series of treatments with high glucose concentration (HG), canagliflozin, compound C, and AICAR, slides were removed, fixed, blocked, incubated with primary antibodies at 37 °C for 2 hours, and then incubated with the secondary antibody and DAPI. Samples were subjected to immunofluorescence microscopy for analysis.

### 2.8 EdU assay

MC3T3-E1 cells were incubated with 10 μM EdU (Beyotime, China) for 2 h at 37 °C. Cell climbing slices were removed, and the detection of EdU was performed according to the instructions of the EdU kit (Beyotime, China). Briefly, after fixation, cells were incubated with click reaction buffer (consisting of CuSO4, Azide 555, and Click Additive Solution) and finally counterstained with DAPI. The EdU-positive cells were quantified using Image J software.

### 2.9 Western blotting analysis

Proteins from the cells were extracted using conventional methods. Then, proteins were separated using 10% or 12% SDS-PAGE and transferred to PVDF membranes. The membranes were incubated with rabbit anti-RUNX2 antibody (1:1000, Abcam, USA, ab 236639), rabbit anti-p-AMPK antibody (Thr172) (1:1000, Cell Signaling Technology, USA, CST 2535), rabbit anti-AMPKα antibody (1:1000, Cell Signaling Technology, USA, CST 2532), and rabbit ALP antibody (Immunoway Biotechnology Company,YT 5375) at 4 °C overnight and then incubated with HRP-conjugated secondary antibody (Invitrogen, USA) for 1 h. Bands were visualized using the BioRad imaging system.

### 2.10 Alkaline phosphatase staining

Alkaline phosphatase staining was performed on the 7th day during differentiation by the BCIP/NBT alkaline phosphatase color development kit (Beyotime, China) according to the manufacturer’s instructions.

### 2.11 Alkaline phosphatase activity

ALP activity was measured after removing the differentiation culture medium on the 7th day. Two parts of the same cell lysate were measured for protein concentration and ALP activity. The protein concentration was determined using a BCA protein assay kit (Beyotime, China). ALP assays were performed according to the instructions of the ALP kit (Jiancheng, Nanjing, China). The final relative ALP activity was calculated by the formula: U/gprot= (test OD405 nm value-blank OD405 nm value)/(standard OD405 nm value-blank OD405 nm value)*the concentration of the standard sample/the protein concentration of the sample.

### 2.12 ELISA

The plasma of the mouse was diluted to a ratio of 1:3. A mouse osteocalcin ELISA kit (Jianglai, Biological Company) was used to measure osteocalcin levels in plasma according to the manufacturer’s instructions.

### 2.13 Flow cytometry

Cells were resuspended and fixed in 70% precooled ethanol overnight, and then washed and incubated for 30 min in 0.1% sodium citrate containing RNase (10 μg/ml) and 50 μg/ml propidium iodide (PI). Each sample contained over 20,000 events collected in a single-cell gate. FlowJo (RRID: SCR_008520) software was used to analyze the distribution of cells in G1, S, and G2/M phases of the cell cycle.

### 2.14 Alizarin red staining

After 14 or 21 days of osteogenic differentiation, cells were fixed with 4% polyoxymethylene for 20 minutes and stained with Alizarin red S (Beyotime, China) for 1 hour. Calcium nodules were counted under the microscope.

### 2.15 Molecular docking

Molecular docking was carried out using AutoDock Tool 1.5.6 (RRID: SCR_012746) software. Crystal structures of AMPK were uploaded from the Protein Data Bank (PDB) internet. PyMOL (RRID: SCR_000305) software was used to visualize the results and create 3D images.

### 2.16 Statistical analysis

Data are presented as the mean ± SD. The paired *t* test was used to compare the two groups before and after the intervention, and one-way ANOVA was used to compare multiple groups. GraphPad Prism (RRID: SCR_002798) was used to handle the original data, and statistical significance was defined as *P*< 0.05.

## 3 Results

### 3.1 Body weight, blood glucose and bone parameters

**Figure 2:**
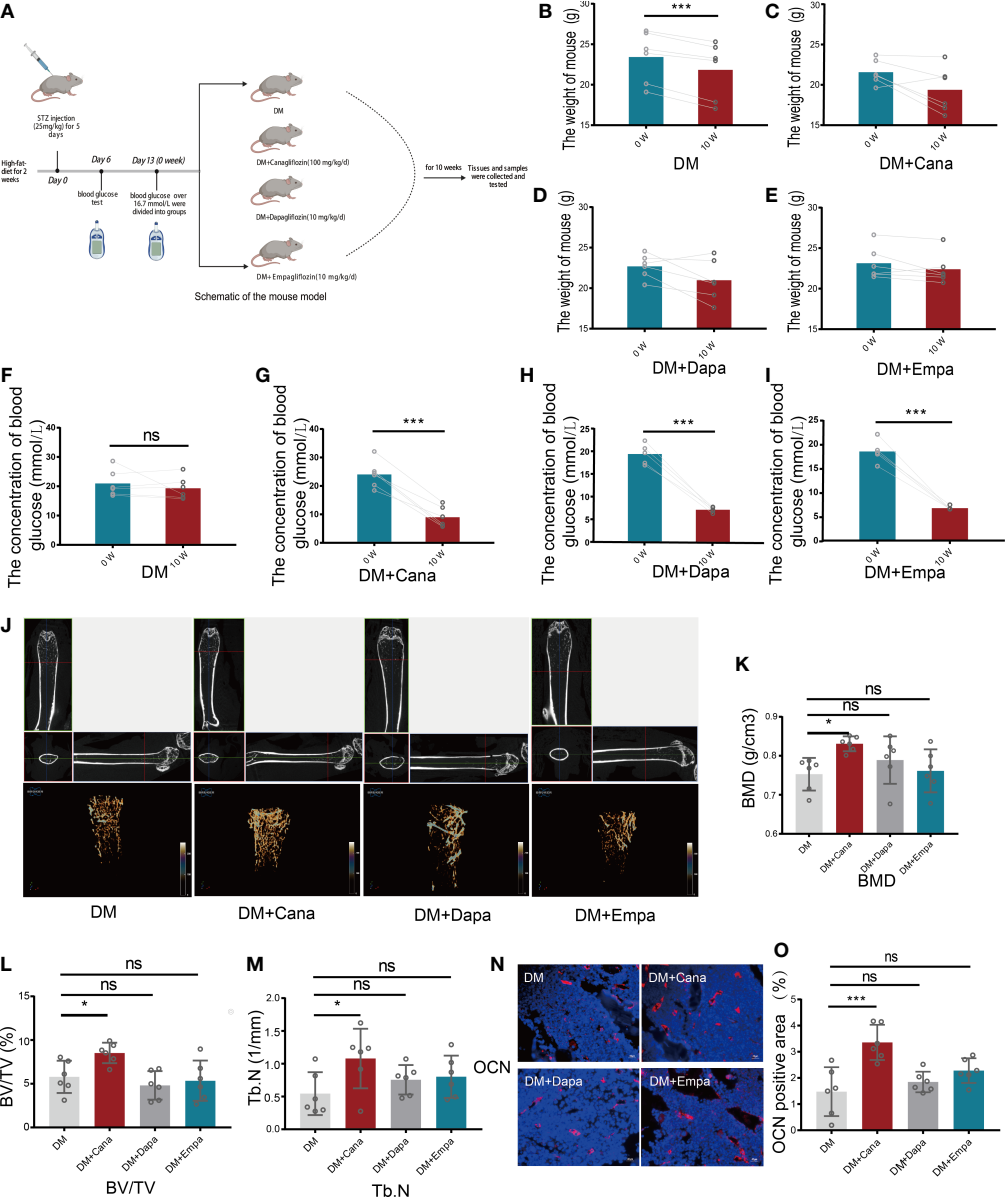
Body weight, blood glucose and bone parameters. **(A)** Schematic of a mouse model (Created with BioRender.com). **(B)** Changes of body weight for DM group (n=6). **(C)** Changes of body weight for DM+Cana group (n=6). **(D)** Changes of body weight for DM+Dapa group (n=6). **(E)** Changes of body weight for DM+Empa group (n=6). **(F)** Changes of blood glucose for DM group (n=6). **(G)** Changes of blood glucose for DM+Cana group (n=6). **(H)** Changes of blood glucose for DM+Dapa group (n=6). **(I)** Changes of blood glucose for DM+Empa group (n=6). **(J)** Representative 2D images of mouse femoral tissue in x-y, y-z, x-z directions above and representative 3D images of mouse femur behind for 4 groups. **(K)** BMD value for 4 groups. **(L)** BV/TV for 4 groups. **(M)** Tb.N for 4 groups. **(N, O)** Expression of OCN in different groups *in vivo* (n=6). *P < 0.05; ***P < 0.001; ns, not statistically significant. The statistical differences were calculated by pair *t* test or one-way ANOVA test.

A schematic representation of the animal model is shown in **Figure 2A**. The changes in general characteristics (body weight and blood glucose) are shown in **Figures 2B–I**. The blood glucose level was significantly decreased in the three antihyperglycemic groups (*P*<0.001), but there was no difference in the change in body weight compared to that of the DM group after SGLT2 inhibitors therapy. However, for the DM without antihyperglycemic therapy group, the body weight was significantly decreased (*P*<0.001), but the blood glucose level was not significantly changed.

Femoral tissues in all groups (DM, DM+Cana, DM+Dapa, and DM+Empa) were analyzed by micro-CT. Only in the DM+Cana group did the trabecular bones have a complete bone structure and a neat arrangement after treatment with SGLT2 inhibitors through micro-CT examination (**Figure 2J**). The bone-related parameters: bone mineral density (BMD), bone volume per tissue volume (BV/TV), and trabecular number (Tb. N) were all significantly increased in the DM+Cana group compared with the DM group (*P*<0.05) (**Figures 2K–M**). However, this phenomenon was not observed in the DM+Daga or DM+Empa group.

To further verify our finding, the expression of OCN, a widely accepted bone formation and remodeling marker, was tested with immunofluorescence staining (**Figures 2N, O**). The rate of positive expression of OCN was significantly higher in the DM+Cana group than in the DM group (*P*<0.001). However, there were no statistically significant differences in the DM+Daga or DM+Empa groups compared with the DM group.

### 3.2 Effects of canagliflozin on the proliferation and viability of MC3T3-E1 cells

**Figure 3:**
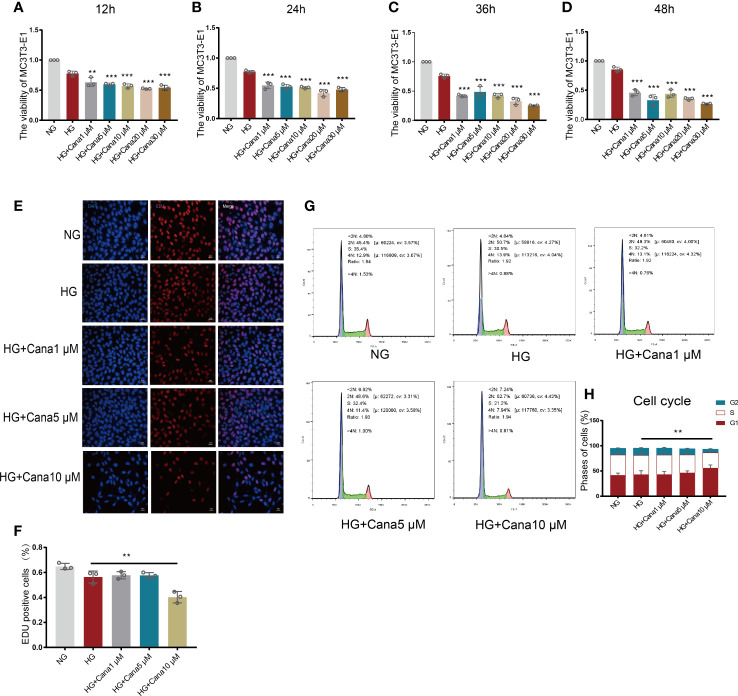
Effects of canagliflozin on the proliferation and viability of MC3T3-E1 cells. **(A–D)** The viability of MC3T3-E1 cells in NG and HG treated with canagliflozin in concentration Gradient for 12 h, 24 h, 36 h, 48h. **(E, F)** Proliferative activity was measured by EDU assay and quantified. **(G, H)** Cell cycle measured by flow cytometry and analyzed by FlowJo software. **P < 0.01, ***P < 0.001 compared with HG group. Data were analyzed using one-way ANOVA for three independent experiments.

Based on the above findings, gradient treatment of canagliflozin was performed on MC3T3-E1 cells under NG or HG conditions. A CCK8 assay was used to test cell viability (**Figures 3A–D**). Cell viability began to be impeded by preconditioning with 1 μM canagliflozin for 12 h under HG compared with the HG-treated only group (*P*<0.01). The EdU assay showed that cell proliferation was impeded at a concentration of 10 μM (**Figures 3E, F**) (*P*<0.01). There was no inhibitory effect on cell proliferation when the concentration of canagliflozin was lower than 5 μM. With flow cytometry, the cell cycle profile was evaluated using PI staining, and the result was consistent with the above findings (**Figures 3G, H**). Canagliflozin at ten micromolar arrested MC3T3-E1 cells in G1 phase and prevented them from entering S phase, where DNA synthesis occurs (*P*<0.01).

### 3.3 Alleviation of high glucose-induced cytotoxicity by canagliflozin during the differentiation of osteoblastic MC3T3-E1 cells

**Figure 4:**
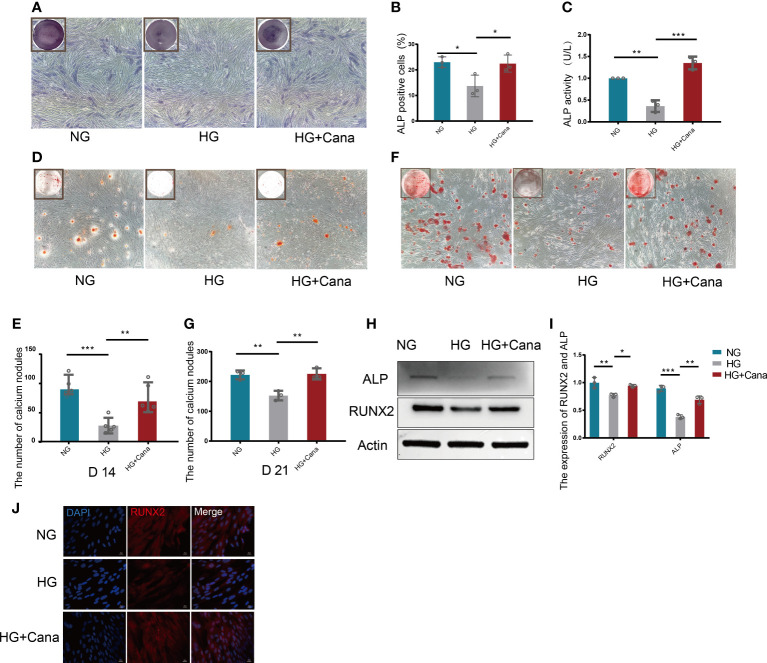
Alleviation of high glucose-induced cytotoxicity by canagliflozin during the differentiation of osteoblastic MC3T3-E1 cells. **(A, B)** ALP staining and were quantified under NG or HG on the 7th day during differentiation. **(C)** ALP activity was measured under NG or HG on the 7th day during differentiation at an absorbance of 405nm. **(D, E)** Alizarin Red S under NG or HG on the 14th day during differentiation and the number of calcium nodules was calculated under the microscope. **(F, G)** Alizarin Red S under NG or HG on 21st day during differentiation and the number of calcium nodules was calculated under the microscope. **(H, I)** Expression of ALP and RUNX2 (on the 3rd day during differentiation) and quantified. **(J)** Expression of RUNX2 (on the 3rd day during differentiation) and captured the picture by fluorescence microscopy. (A concentration of 5 μM canagliflozin was used). *P < 0.05; **P < 0.01; ***P < 0.001; Data were analyzed using one-way ANOVA for three independent experiments.

ALP staining is shown in **Figures 4A, B**. HG treatment decreased the percentage of ALP positive cells compared with that in the NG group (*P*<0.01), and the decreased percentage of ALP positive cells could be improved in the HG+Cana group (*P*<0.001). The trend of ALP activity corresponded to the above result (**Figure 4C**). To verify whether canagliflozin affects the formation of calcium nodules, we performed Alizarin red staining experiments on the 14th and 21st days during the differentiation of MC3T3-E1 cells (**Figures 4D–G**). The number of calcium nodules the HG group was less than that in the NG group on the 14th and 21st day during the differentiation of MC3T3-E1 cells. Notably, the HG+Cana group had increased formation of calcium nodules on the 14th and 21st days during the process of cell differentiation under HG. The results above were verified by western blotting (**Figures 4H–J**). Consistently, the expression of ALP, an early marker of osteoblast differentiation, was increased in the HG+Cana group. Furthermore, the expression of RUNX2, an important bone formation marker, was measured by immunofluorescence staining and western blotting, which showed the same expression trend as that of ALP.

### 3.4 Canagliflozin promotes differentiation of osteoblastic MC3T3-E1 cells partially through the AMPK/RUNX2 pathway under HG conditions *in vitro*

**Figure 5:**
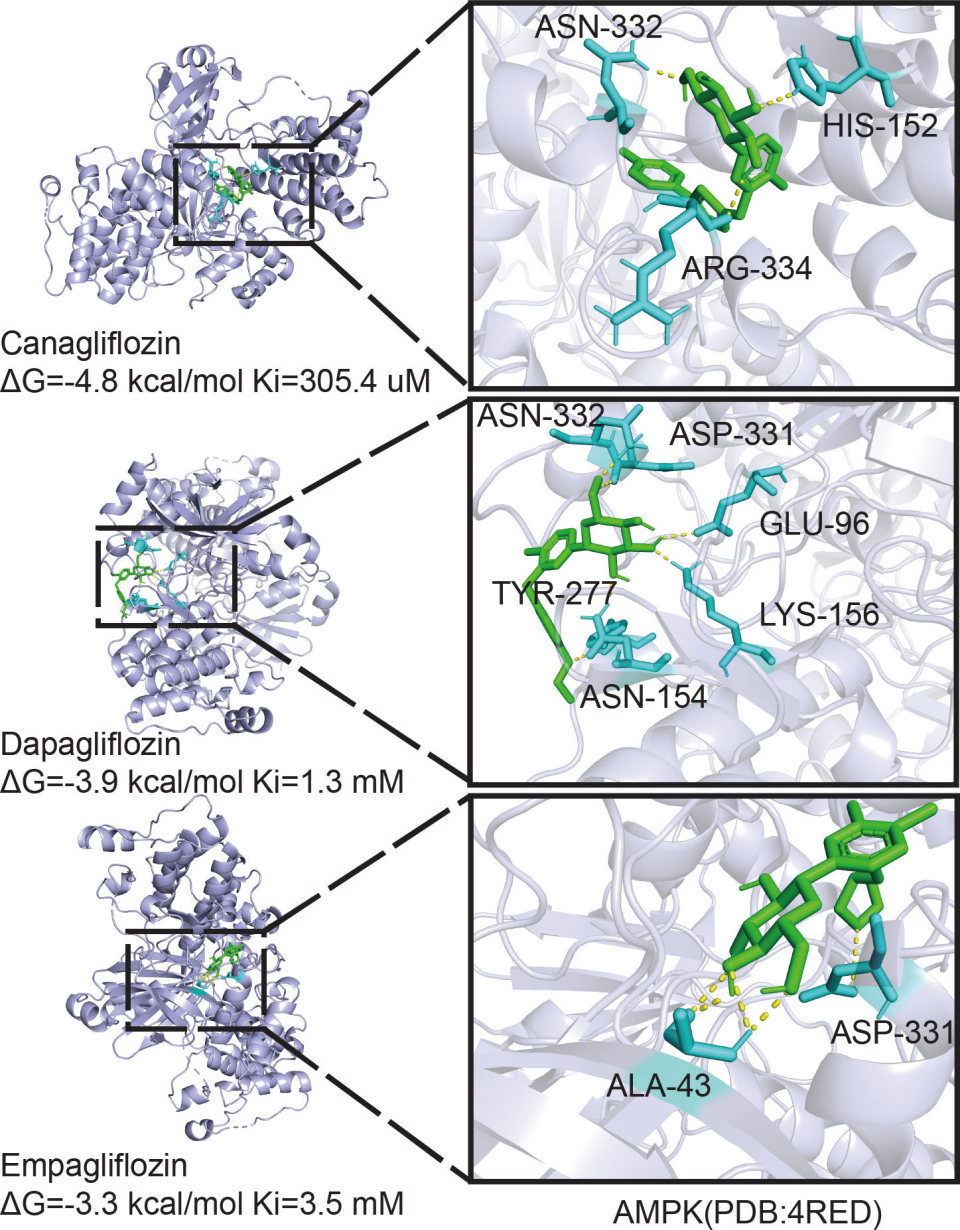
Molecular docking analysis. Phosphorylation AMPKα (Thr172) structure download from PDB database binding with canagliflozin, dapagliflozin, and empagliflozin respectively, the image on the right is enlarged of the left.

**Figure 6:**
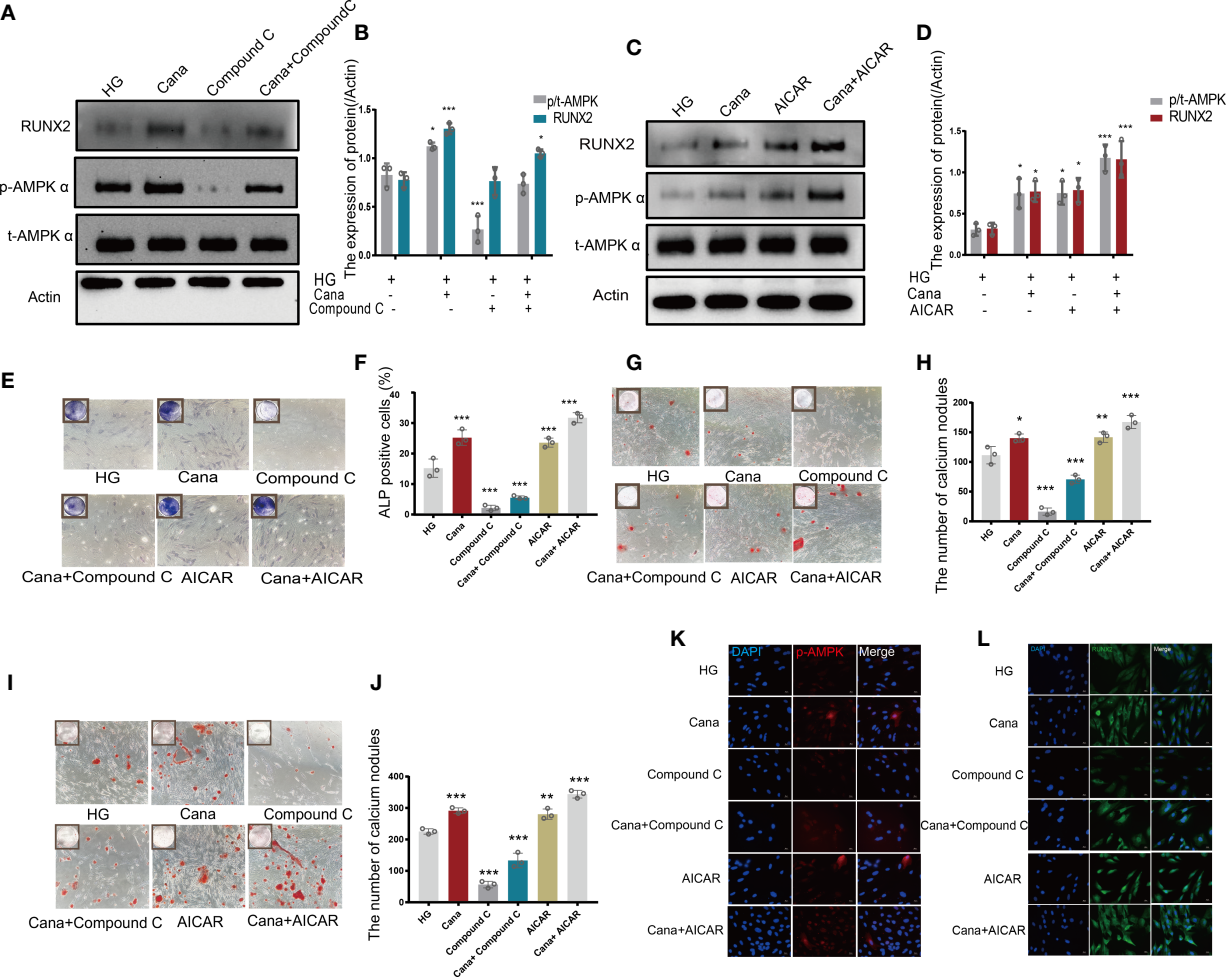
Canagliflozin promotes differentiation of osteoblastic MC3T3-E1 partially through the AMPK/RUNX2 pathway under HG. **(A, B)** Expression of t-AMPK, phosphorylation AMPKα (Thr172), and RUNX2 detected under HG (with or without canagliflozin and compound C) on the 3rd day during differentiation and quantified. **(C, D)** Expression of phosphorylation AMPKα (Thr172) and RUNX2 examined under HG (with or without canagliflozin and AICAR) on the 3rd day during differentiation and quantified. **(E, F)** ALP staining under HG (with or without canagliflozin, compound C, and AICAR) on the 7th day during differentiation and quantified. **(G, H)** Alizarin Red S under HG (with or without canagliflozin, compound C, and AICAR) on the 14th day during differentiation the number of calcium nodules was calculated under the microscope. **(I, J)** Alizarin Red S under HG (with or without canagliflozin, compound C, and AICAR) on the 21st day during differentiation and the number of calcium nodules was calculated under the microscope. **(K)** Expression of phosphorylation AMPKα (Thr172) under HG (with or without canagliflozin, compound C, and AICAR) (on the 3rd day during differentiation) and captured the picture by fluorescence microscopy. **(L)** Expression of RUNX2 under HG (with or without canagliflozin, compound C, and AICAR) (on the 3rd day during differentiation) and captured the picture by fluorescence microscopy. (The concentration of canagliflozin was 5 μM, compound C was 1 μM, AICAR was 10 μM). *P< 0.05, **P < 0.01, ***P < 0.001 compared with HG group. Data were analyzed using one-way ANOVA for three independent experiments.

Owing to the different molecular structures among these three inhibitors, PyMol software was used to perform molecular docking visualization to compare the binding of SGLT2 inhibitors (canagliflozin, dapagliflozin, and empagliflozin) to AMPK phosphorylated at threonine 172 (PDB:4RED) (http://www.rcsb.org). The binding energy between canagliflozin and p-AMPK (ΔG=-4.8 kcal/mol) was the smallest compared with those of dapagliflozin (ΔG=-3.9 kcal/mol) and empagliflozin (ΔG=-3.3 kcal/mol) (**Figure 5**). Therefore, we next explored the expression of RUNX2 after treatment with the inhibitor and the activator of AMPK. Western blotting showed that when p-AMPK was inhibited by compound C, the expression of RUNX2 was hampered, and canagliflozin partly reversed this effect in the Cana+Compound C group (**Figures 6A, B**). The quantities of RUNX2 increased, followed by an increase in p-AMPK/t-AMPK (**Figures 6C, D**). As shown in **Figures 6E, F**, ALP staining on the 7th day during differentiation and Alizarin red staining during differentiation on the 14th or 21st day showed a consistent trend with the above results (**Figures 6G–J**). The expression of p-AMPK and RUNX2 was determined using immunofluorescence staining (**Figures 6K, L**), and the value of the fluorescence intensity of RUNX2 is in accordance with the fraction of p-AMPK (Thr172) augmented in MC3T3-E1 cells.

### 3.5 Improvement of bone microarchitecture in type 2 diabetic mice

**Figure 7:**
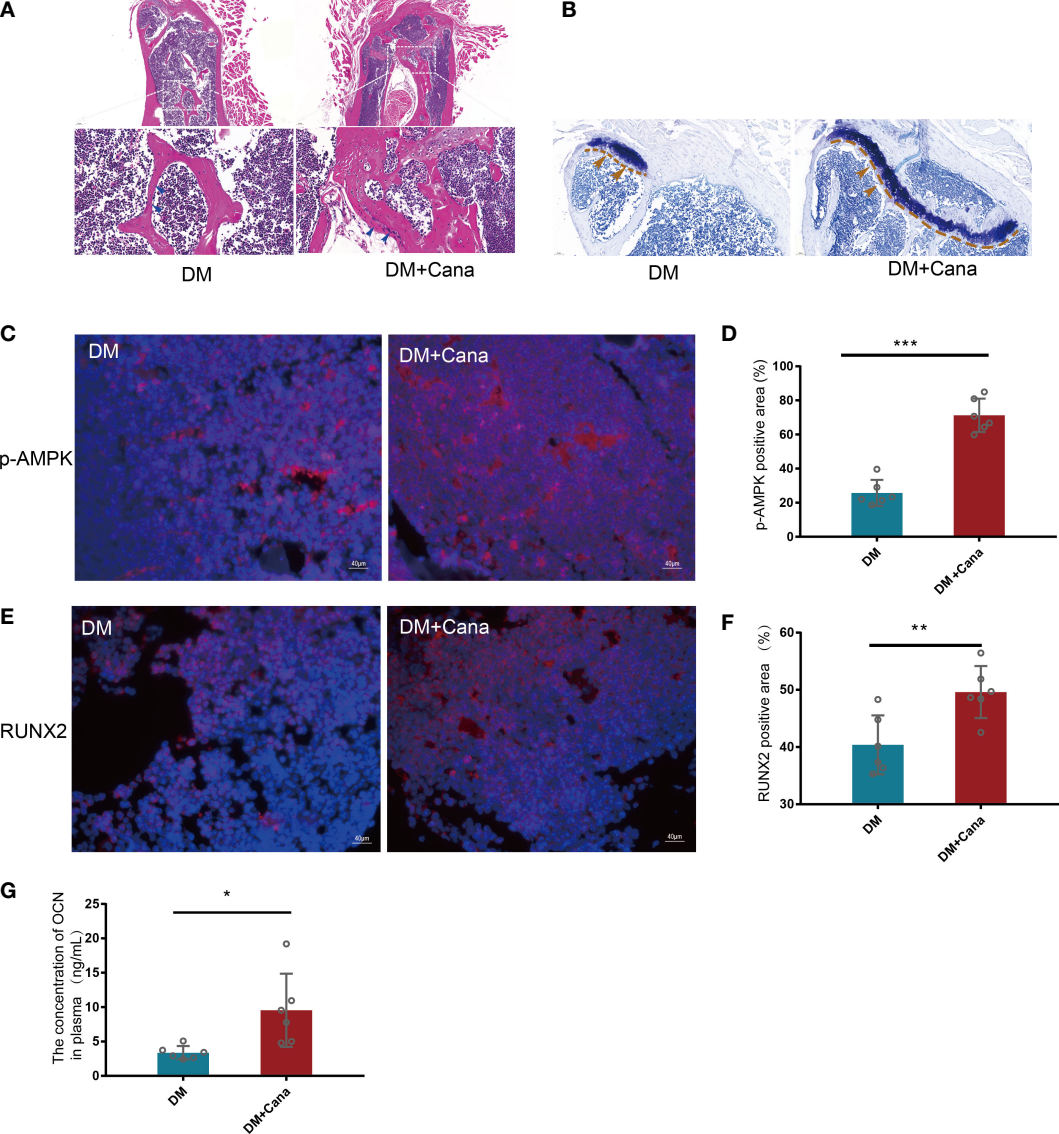
Canagliflozin can improve bone microarchitecture in Type 2 Diabetic Mouse Models. **(A)** HE staining on femoral tissue in DM and DM+Cana groups, the image below is the magnified of the picture above, the bar is in the bottom left corner of the picture. **(B)** Toluidine blue staining on femoral tissue in DM and DM+Cana groups, the bar is in the bottom left corner of the picture. **(C, D)** Expression of phosphorylation AMPKα (Thr172) in DM and DM+Cana groups and quantified. **(E, F)** Expression of RUNX2 in DM and DM+Cana groups and quantified. **(G)** The concentration of OCN in plasma determined by OCN Elisa Kit. *P < 0.05; **P < 0.01; ***P < 0.001; Data were analyzed using *t* test.

*In vivo*, the DM+Cana group had a higher percentage of osteoblasts in trabecular bone than the DM group, according to HE staining (**Figure 7A**). Toluidine blue staining showed that the area of the femoral growth plate in the DM+Cana group was larger than that in the DM group (**Figure 7B**). To prove whether the expression levels of RUNX2 and p-AMPK *in vivo* were consistent with those *in vitro*, immunofluorescence staining was performed (**Figures 7C–F**), and the expression levels of RUNX2 and p-AMPK (Thr172) were higher in the canagliflozin treatment group. The concentration of OCN in plasma in the canagliflozin treatment group was increased compared with that in the DM group (*P*<0.05) (**Figure 7G**).

### 3.6 Inhibition of osteoclast *in vitro and vivo*

**Figure 8:**
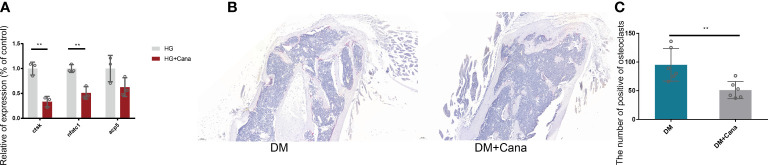
Effect of canagliflozin on osteoclast *in vitro* and *vivo*. **(A)** Relative osteoclastic bone gene expression on osteoclast *in vitro* with or without canagliflozin under HG. **(B)** Trap staining on femoral tissue for DM and DM+Cana groups. **(C)** The quantitative statistics of B). **P < 0.01. Data were analyzed using *t* test for three independent experiments.

**Figure 9:**
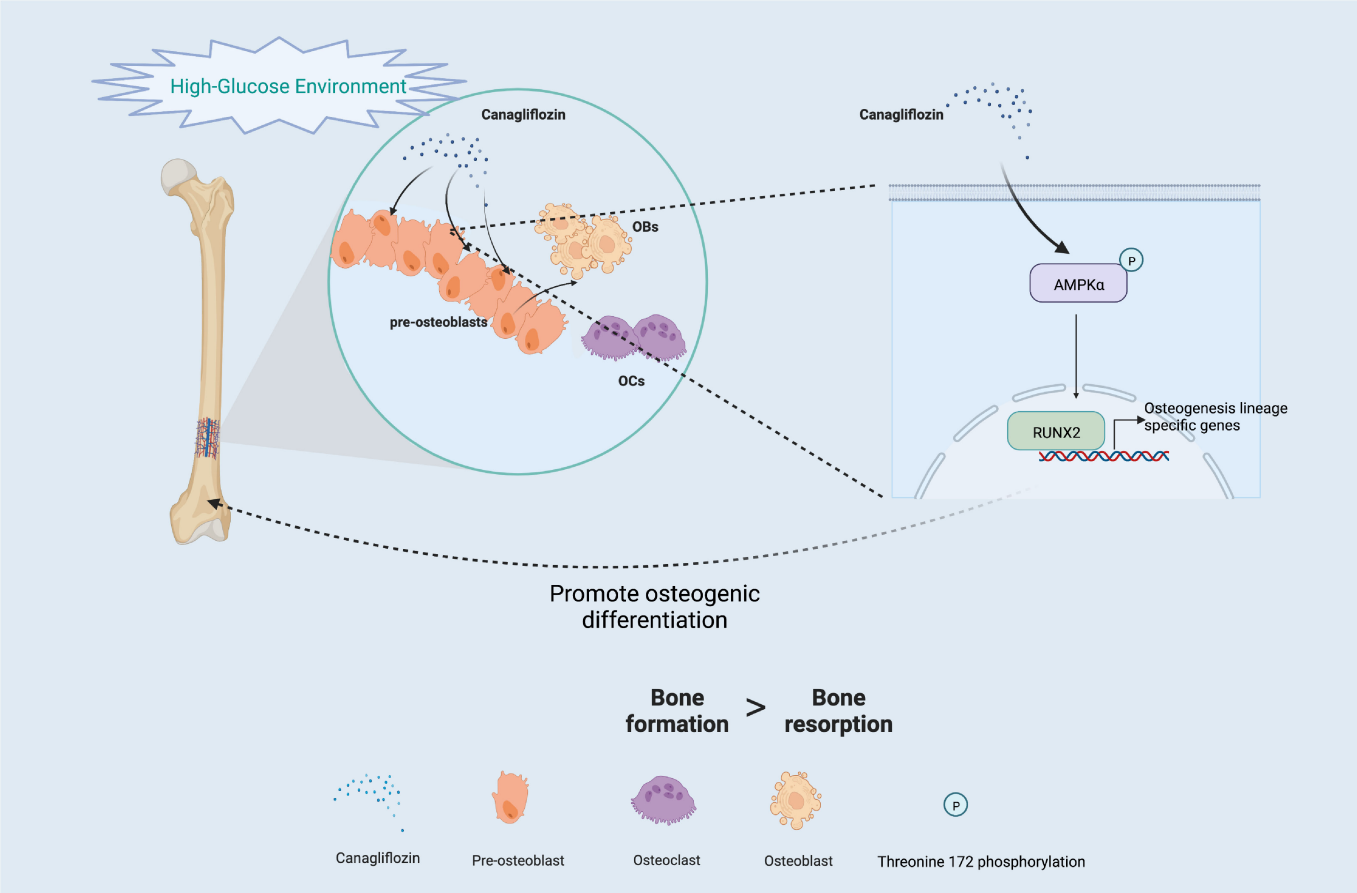
Schematic of the molecular mechanism under HG (Created with BioRender.com).

Another major finding from our study is that canagliflozin hampered osteoclast-related gene expression in osteoclasts *in vitro* (**Figure 8A**). The primer sequences are listed in **Supplementary Table S1**. Trap staining in femoral tissue showed the same results (**Figures 8B, C**), which suggests that canagliflozin is associated with increased bone formation and decreased bone resorption. Inhibition of bone resorption may be an additional route to explore the result of increasing bone mass. In addition, we developed a model to illustrate AMPK-mediated osteoblastic differentiation under HG by inducing RUNX2 expression (**Figure 9**).

## 4 Discussion

Bone metabolic disorders and fracture in T2DM remain a major economic and health burden worldwide. In addition to the benefits of cardiovascular disease, antidiabetic agents were also paid attention to other complications related life span, such as bone fracture. According to recent data on antihyperglycemic agents, metformin is beneficial to bone formation by suppressing osteoblast apoptosis and activating osteogenic differentiation signaling (31, 32). GLP-1 drugs, like liraglutide, showed a significant improvement in BMD, bone microarchitecture, and bone biomechanical markers (5–7). In addition, supplementation with insulin for T2DM patients induced bone regeneration (8). Nevertheless, thiazolidinediones are correlated with osteoporosis and increased fracture risk (33). SGLT2 inhibitors, as monotherapy or adjunct therapy for reversal of hyperglycemia agents, are a revolutionary treatment for patients with diabetes. They provide a novel treatment pattern for this condition, which may have off-target effects in different tissues (34–38). Some studies showed that SGLT2 inhibitors were related to adverse events of bone in the treatment of T2DM (14, 39–41). However, the latest research demonstrated that there was no direct association between SGLT2 inhibitors treatment and the risk of bone fracture, and the treatment even partially prevented bone deficits (41–46). Patients enrolled in the CANVAS study had a history of cardiovascular events, a lower baseline eGFR and a higher baseline diuretic use, and was enrolled in an elderly population. However, our research is based on only type 2 diabetes mellitus. The difference in baseline may explain the discrepancy between our study and the CANVAS study. In addition, canagliflozin could increase osteoid volume/bone volume (OV/BV), osteoid surface/bone surface (OS/BS), BV/TV, and trabecular thickness (Tb. Th) in a type 1 diabetic mouse model (47). However, recent studies have conflicting results regarding the relationship between SGLT2 inhibitors and bone fracture (41–43, 45) and the related mechanism in type 2 diabetes. A subsequent investigation into the possible effect and mechanism led us to seek the functions of SGLT2 inhibitors in bone metabolism. These clinical studies were not standardized because of the difference in regions and populations, and basic studies are desperately needed for clarification of these findings.

Evidence in this study confirmed our hypothesis: first, in addition to its blood glucose-lowering effects *in vivo*, canagliflozin has a distinct effect on the arrangement and number of trabecular bone cells compared with dapagliflozin and empagliflozin. Second, canagliflozin hampered cell proliferation at a concentration of up to 10 μM by impeding the cell cycle, and no proliferation impact was observed when the concentration was below 5 μM in the cell medium under HG. This result suggested that we use 5 μM to explore the potential effect of canagliflozin during the process of osteoblastic MC3T3-E1 differentiation. Third, canagliflozin alleviated high glucose-induced cytotoxicity, possibly partially through the AMPK/RUNX2 pathway. This finding was also preliminarily verified *in vivo*. The evidence we provided in this study supports a protective role for canagliflozin in diabetic bone metabolism through a distinct mechanism, namely, the alleviation of bone metabolic disorders, for the first time.

The present study identifies canagliflozin as a novel activator of osteoblastic MC3T3-E1 differentiation but an inhibitor of MC3T3-E1 proliferation at a concentration of up to 10 μM. The findings of this study appear to differentiate canagliflozin from other SGLT2 inhibitors, such as dapagliflozin and empagliflozin, at pharmacologically relevant concentrations. A previous study showed that canagliflozin increased bone formation and resorption biomarkers in older patients with T2DM (48). A meta-analysis indicated that canagliflozin may show benefits in terms of bone fracture (49).

SGLT2 has recently been reported to be expressed in multiple cells, and SGLT2 functions as an important glucose transporter in these cells (50).AMPK regulates cellular metabolism to maintain energy homeostasis by acting as a physiological energy sensor (51, 52). The AMPK complex has three different subunits, especially the AMPK*α* subunit, which is highly expressed in bone tissue as well as in several bone cell lines. Phosphorylation of AMPK at threonine 172 is required for the activation of the α catalytic subunit by upstream kinases (53). The activation of AMPK was implicated in previous studies as the mechanism by which empagliflozin and dapagliflozin benefited mitochondria (54–56). However, only cells treated with canagliflozin displayed AMPK phosphorylation (57). Therefore, we concluded that the binding energy between canagliflozin and p-AMPKα (Thr172) is the smallest compared with empagliflozin and dapagliflozin by performing molecular docking. The lower the binding energy is, the more stable the structure and the easier it is for the molecules to interact with each other. The difference between these three inhibitors may contribute to the molecular structure. We observed that canagliflozin induces the expression of ALP and RUNX2 and increases ALP activity in osteoblastic MC3T3-E1 cells. The induction of RUNX2 by canagliflozin requires activating AMPK activity by inducing AMPK phosphorylation under HG.

Interestingly, inhibition of osteoclast gene expression was still observed on osteoclasts *in vitro* at the therapeutic dose, that is, this bidirectional regulation deserves further exploration. Although our study had significant discoveries in bone differentiation, the functions of canagliflozin in bone resorption remain to be seen. In this study, we used a 100 mg/kg/d dose for animals and a 5 μM concentration for cells, and the dose effects of canagliflozin may be studied in future trials.

## 5 Conclusion

In summary, our results indicate that AMPK/RUNX2 signaling regulates osteoblastic differentiation prominently. These results provide evidence for future studies on the AMPK signaling pathway in bone metabolism, and canagliflozin may exhibit a bidirectional regulative effect between the metabolic balance of osteoblast and osteoclast. These pleiotropic effects of canagliflozin on bone metabolism may benefit for bone formation and inhibit bone absorption, particularly in patients with T2DM and suggesting additional benefits of canagliflozin.

## Data availability statement

The datasets presented in this study can be found in online repositories. The names of the repository/repositories and accession number(s) can be found in the article/**Supplementary Material**.

## Ethics statement

The animal study was reviewed and approved by The ethics committee of Chongqing University Central Hospital.

## Author contributions

PS and TC: research conducted and original draft preparation. SR, XD, and BD: data visualization and analysis. DA: supervision. YM and WD: project designed and writing-reviewing. The final manuscript was read by all authors. All authors contributed to the article and approved the submitted version.
