# Supplementary material for: Canagliflozin promotes osteoblastic MC3T3-E1 differentiation via AMPK/RUNX2 and improves bone microarchitecture in type 2 diabetic mice
Source: Front Endocrinol (Lausanne). 2022 Dec 16;13:1081039. doi: 10.3389/fendo.2022.1081039 (PMC9800613; doi:10.3389/fendo.2022.1081039)
Supplement: Supplementary file 1 [file Table_1.docx]

Supplementary Material

# Supplementary Table

## Supplementary Table S1.The primer sequences are as follows.

| **Primer** | **Sequence(5'→3')** | **Base count (n)** |
| --- | --- | --- |
| mouse-Nfatc1-F | GCTCCTGTGTTCCGTGTTCTGTC | 23 |
| mouse-Nfatc1-R | GCATCCATTCATTAGCATCGGTGTTC | 26 |
| mouse-Ctsk-F | GGATATGCTCTCTTGGCTCGGAATAAG | 27 |
| mouse-Ctsk-R | GGCTGGCTGGAATCACATCTTGG | 23 |
| mouse-Acp5-F | TGCGACCATTGTTAGCCACATACG | 24 |
| mouse-Acp5-R | CACACCGTTCTCGTCCTGAAGATAC | 25 |
| mouse-Actin-F | ACCCCGTGCTGCTGACCGAG | 20 |
| mouse-Actin-R | TCCCGGCCAGCCAGGTCCA | 19 |
